# Ultrasonic Treatment of Food Colloidal Systems Containing Oleogels: A Review

**DOI:** 10.3390/gels8120801

**Published:** 2022-12-07

**Authors:** Yuliya Frolova, Varuzhan Sarkisyan, Roman Sobolev, Alla Kochetkova

**Affiliations:** Laboratory of Food Biotechnology and Foods for Special Dietary Uses, Federal State Budgetary Scientific Institution Federal Research Center of Nutrition, Biotechnology and Food Safety, 109240 Moscow, Russia

**Keywords:** ultrasound, gelation, oleogels, emulsions, bigels, food products

## Abstract

The use of oleogels as an alternative to solid fats to reduce the content of saturated and *trans*-isomeric fatty acids is a developing area of research. Studies devoted to the search for methods of obtaining oleogels with given properties are of current interest. Ultrasonic treatment as a method for modifying oleogel properties has been used to solve this problem. The number of publications on the study of the effect of ultrasonic treatment on oleogel properties is increasing. This review aimed to systematize and summarize existing data. It allowed us to identify the incompleteness of this data, assess the effect of ultrasonic treatment on oleogel properties, which depends on various factors, and identify the vector of this direction in the food industry. A more detailed description of the parameters of ultrasonic treatment is needed to compare the results between various publications. Ultrasonic treatment generally leads to a decrease in crystal size and an increase in oil-binding capacity, rheological properties, and hardness. The chemical composition of oleogels and the concentration of gelators, the amplitude and duration of sonication, the cooling rate, and the crystallization process stage at which the treatment occurs are shown to be the factors influencing the efficiency of the ultrasonic treatment.

## 1. Introduction

Dietary fats and oils are an essential part of the diet, necessary for normal body functioning and growth [1]. They are widely used in the food industry due to their physicochemical and functional properties. Fats provide distinctive flavors and aromas, textures, and mouth feels to finished products [2,3], and they are also a source of essential fatty acids and contribute to rapid satiety [1,3]. The desired texture of fat-based food products (margarine, spreads, shortenings, etc.) is achieved through the use of hydrogenated vegetable oils containing triglycerides with *trans* fatty acids formed during hydrogenation [4]. Since the consumption of *trans* fatty acids isomers is associated with negative health effects, various approaches such as chemical and enzymatic transesterification, the use of fat substitutes, and oleogelation have been developed. This leads to the production of fats which do not contain *trans*-isomers, but which have the desired rheological characteristics [2,5,6].

**Figure 1:**
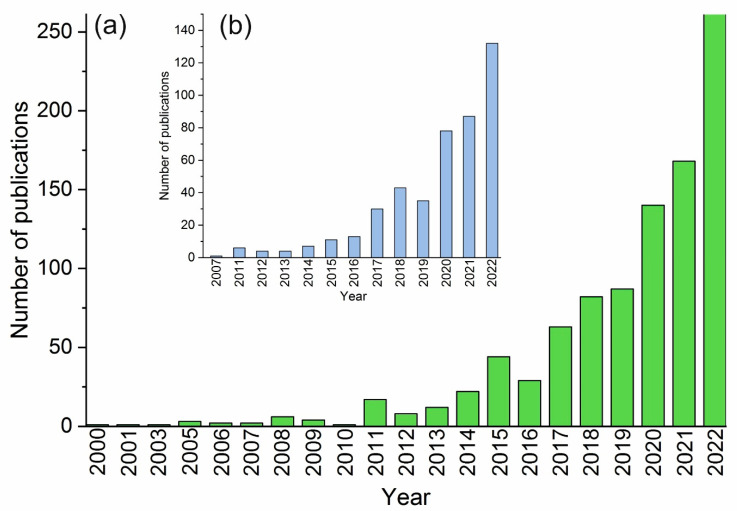
The number of articles on oleogelation topic per year; (**a**) Scopus database search using the keyword “oleogels”; (**a**,**b**) Scopus database search using the keyword “oleogels” and “food”; (actualization date 18 October 2022).

Over the past 20 years, there has been a significant increase in the number of studies on oleogels as an alternative to solid fats. The search for publications was carried out using the Scopus bibliographic and abstract database using the keyword “oleogels” in the thematic fields (article title, abstract, and keywords). The results were refined according to the publication date from 2000 to 2022. In total, 954 publications were identified (with the actualization date of 18 October 2022) (Figure 1a). Publications cover different fields of knowledge, with the leading ones being Agricultural and Biological Sciences (27.8%), Chemistry (20.3%), and Chemical Engineering (13%). The search for the keywords “oleogels” and “food” revealed 459 publications (Figure 1b).

The rapid growth in the number of publications in the field of oleogel studies indicates the relevance of this research direction. Numerous studies have investigated different component compositions of oleogels [7], methods of oleogel production [8,9], and their influence on the microstructural, thermal, rheological, and textural properties, as well as on their oxidative stability [10,11,12]. The study of changes in these properties provides data on the factors affecting the properties of finished oleogels, as well as the possibility of regulating the finished products containing oleogels, which may alsodepending on the technological tasks and given the desired properties. Also oleogels may have bioactive properties due to the content of additional ingredients, depending on the technological tasks and given the desired properties.

This review is focused on the use of ultrasonic treatment as a way to modify the properties of oleogels, including emulsion oleogels. It aims to systematize and summarize the current knowledge in the field, identifying the gaps for a complete assessment of the effect of ultrasonic treatment on the properties of oleogels depending on various factors, as well as identifying the vector of development of this area of the food industry.

## 2. Oleogels in Food Products

The possibility of creating oleogels with tailored properties and further prediction of their behavior under specific technological conditions is of substantial interest to many industries, in particular the food industry [13]. Oleogels are structured edible oils in which edible oils (up to 90%) are used as a dispersion medium and low or high molecular weight gelling agents as a dispersion phase [14]. The composition of oleogels is often diverse and can vary both in the dispersion medium [15,16,17] and the dispersed phase [18,19], which in turn affects the final properties of the oleogels. Usually, canola oil [19], sunflower or linseed oil [16,20], and fish oil [21] are used as dispersion mediums. The use of different oils, including their combinations, allows for the regulation of the fatty acid composition of both oleogels and the products containing them. By using one type of gelling agent with different oils, it is possible to obtain oleogels with various textural characteristics [17]. The choice of the dispersed phase is also diverse and includes compounds, both with low molecular weight such as monoglycerides [22], sterols [23], waxes or their fractions [24] and with high molecular weight—ethyl cellulose [25], methylcellulose or hydroxypropyl methylcellulose [26], etc. Despite the existence of many potential gelators, only a few of them can be used for oil structuring in the food industry.

**Figure 2:**
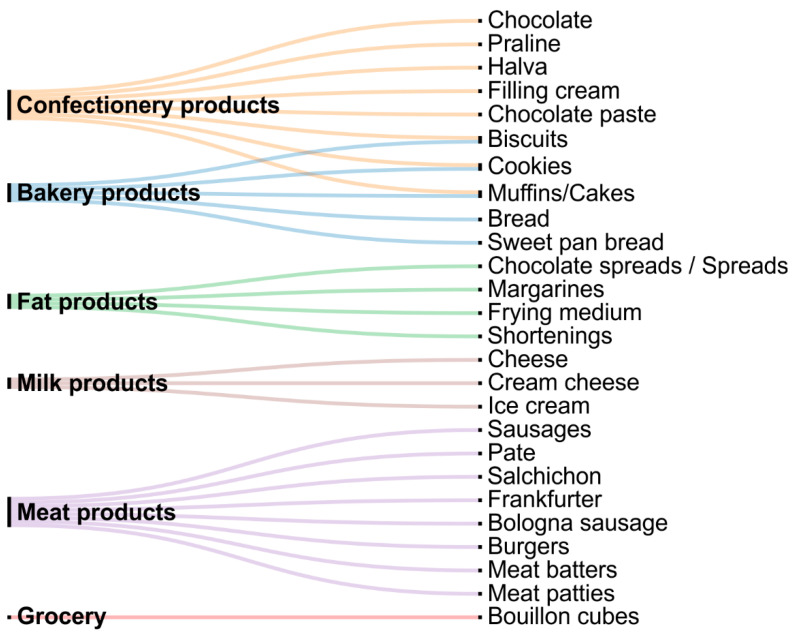
Application of oleogels and emulsion gels in various categories of food products.

Figure 2 summarizes the use of oleogels and emulsion gels in various areas of the food industry as an alternative to solid fats.

Figure 2 shows that the use of oleogels, including emulsion oleogels, as a substitute for solid fat in various food products is widely studied. Among the variety of food products studied are muffins and cookies (muffins/cakes) [27,28,29,30], filling creams [31,32,33], margarines [34,35,36], shortenings [37,38], sausages [39,40,41], frankfurters [42,43,44,45,46], pates [47,48,49,50], burgers [51,52,53,54], etc. These food categories are characterized by high fat content and high consumer demand. Currently, products containing emulsion oleogels are being studied, as well as the use of oleogels in the composition of food products, for example burgers [55], frankfurters [43], margarines [56], shortenings [57], chocolate spreads [58], and cookies [59]. Studies show that oleogels may successfully replace the fat component while maintaining the textural and organoleptic characteristics of the finished products. Depending on the type of product and production technology, it is possible to partially or completely replace the traditionally used fat component with oleogel. Given that liquid vegetable oils with a high content of mono- and polyunsaturated fatty acids are usually used as a dispersion medium in the production of oleogels, the replacement of solid fats with oleogels and emulsion oleogels will allow the fatty acid composition of food products to be regulated.

## 3. Application of Ultrasonic Treatment in the Food Industry

**Figure 3:**
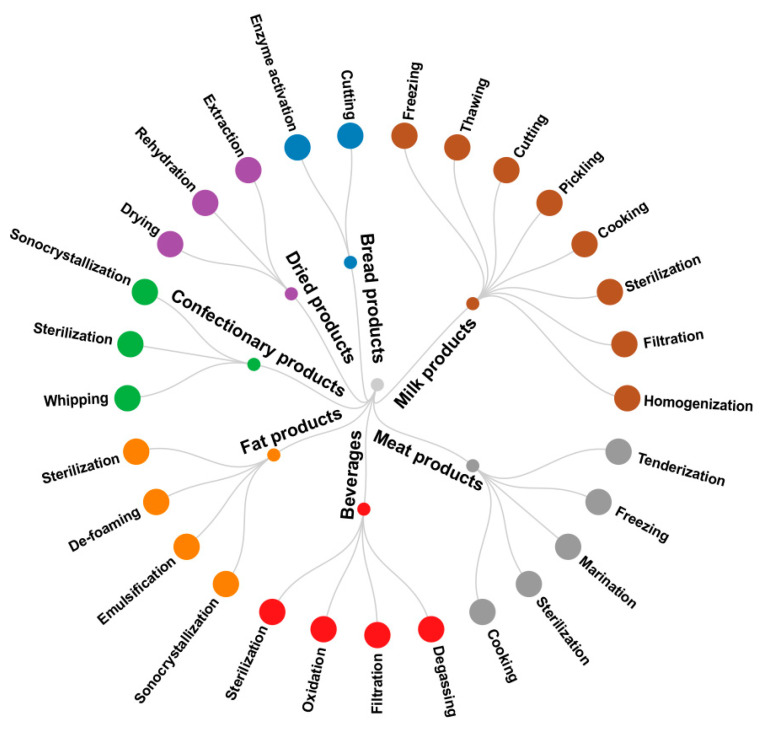
Application of ultrasonic treatment in various branches of the food industry.

Ultrasonic processing is widely used in various branches of the food industry [60,61]. Ultrasound is a form of energy generated by acoustic waves, which can propagate in gases, liquids, or solids [62]. Depending on the technological tasks in the food industry, ultrasound is used with different intensities—from 20 to 2400 kHz [63,64]. Two types of ultrasonic treatment are used—low-intensity and high-intensity. The principle of low-intensity ultrasound (diagnostic waves) is the use of interactions between the substance and high-frequency sound waves, which allows for information to be obtained about the structure, physical condition, or the composition of the food through which it spreads [65]. This type of ultrasound is an inexpensive, fast, simple, accurate, and effective tool for non-destructive analysis and control of the properties of food during processing and storage, such as in quality control of freshly grown fruits and vegetables [66], dairy products and cereals [67], bread, honey, meat, and fish products [68]. High-intensity ultrasound can affect several properties of food: physical, biochemical, and mechanical. Figure 3 shows the branches of the food industry, where ultrasonic processing is used.

There has been a recent growing interest in the use of high-intensity ultrasound. It is used for emulsification [69], the extraction of biologically active components from plants [70,71], the inactivation of microorganisms, and influence on enzyme activity [72]. A recent study [73] shows that ultrasound can also be used to change the crystallization behavior of fats and improve texture and elasticity. There are reports [74] that the use of ultrasound in some food systems leads to a significant improvement in the absorption and bioavailability of omega-3 fatty acids. It is caused by reducing a droplet’s size, which provides a larger surface area to increase lipase activity on the surface and interfacial layer of oil droplets [75]. The application of this type of ultrasound during crystallization is called sonocrystallization. It has considerable potential to improve the crystallization process [76]. A new direction in the field of ultrasonic treatment is its application in the development of oleogels [73,77]. Through the ultrasonic treatment of oleogels, it is possible to vary the size of the crystals formed, which in turn leads to a change in functional and technological properties, such as oil-binding capacity, and textural properties [73,78]. At the same time, one study [79] shows that the effectiveness of ultrasound depends on the cooling rate of oleogels and, under improper conditions, the effect of ultrasound exposure can be minimized.

## 4. Ultrasonic Treatment of Food Colloidal Systems Containing Oleogels

**Figure 4:**
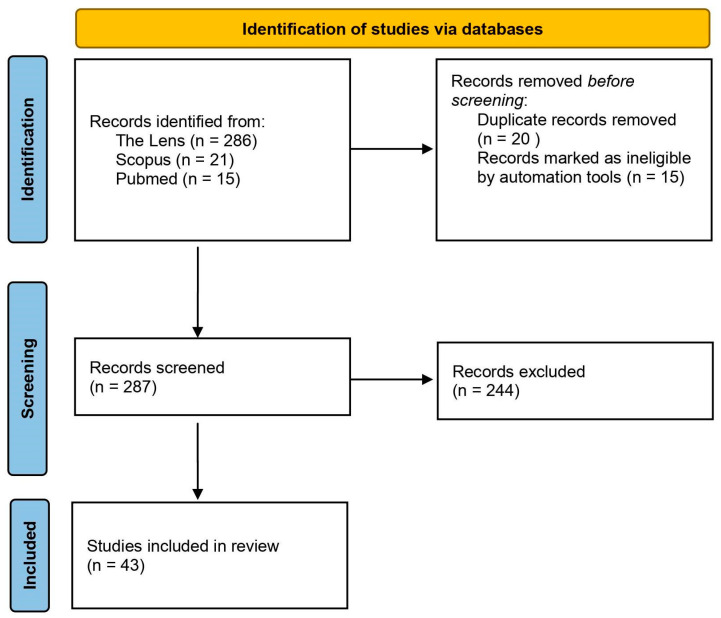
PRISMA flow diagram of the study.

It is known that the properties of oleogels largely depend on processing conditions, such as cooling rate [80], shear forces [81], annealing [82], or ultrasonic processing [83]. In this review, we analyzed published works on the use of ultrasonic treatment in obtaining oleogels and the study of its effect on the properties of the resulting systems. To summarize the data and identify vectors of development in the field of ultrasonic treatment of oleogels, the literature search was performed using the keywords and phrases “oleogels” AND (“ultrasound” OR “ultrasonic”) on the databases Scopus, Lens, and PubMed in the fields of the topic (article title, abstract, keywords), with the date of actualization being 18.10.2022. The algorithm described in [84] was used to analyze publications in this work. Figure 4 presents a flowchart describing the course of the analysis of publications.

Publications for the analysis were selected based on the following inclusion and exclusion criteria. 

Inclusion criteria: (1) open-access publications, (2) inclusion of original research articles only, (3) publications in English.

Exclusion criteria: (1) books, reviews, abstracts, congresses and conferences, systematic reviews, short communications, (2) repeated studies, (3) articles where full text is not available (abstract only). 

**Figure 5:**
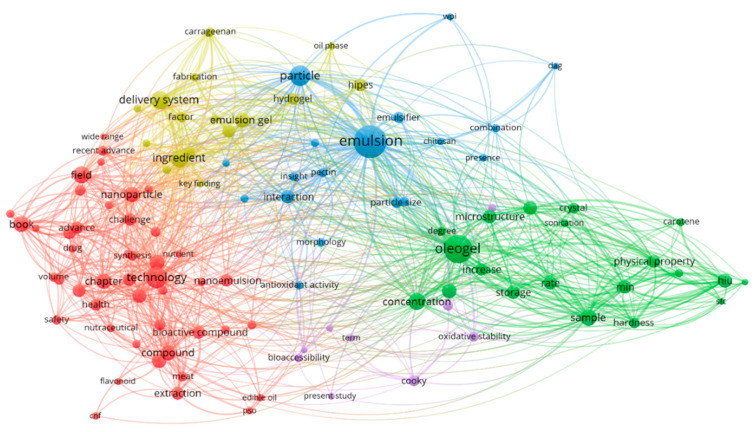
The network visualization of the publications (patents excluded).

A total of 43 publications were selected for analysis according to the inclusion/exclusion criteria. According to the analysis of the publications for the last five years, an increase in the number of publications devoted to the influence of ultrasonic treatment on the properties of oleogels has been observed. Network visualization of publications was obtained using the VOSviewer version 1.6.18 (Van Eck and Waltman, Leiden University, Leiden, The Netherlands) (Figure 5).

Five clusters were identified as a result of the analysis of the publications. The clusters include studies of the use of ultrasound to produce oleogels containing biologically active substances, studies of the effect of ultrasonic treatment on the properties of oleogels, studies of various emulsion systems containing oleogels obtained using ultrasonic treatment, and studies of food products containing oleogels obtained using ultrasonic treatment. According to the selected inclusion/exclusion criteria and results of cluster analysis, publications, including patents, were selected for detailed analysis. The following publication ranking was proposed:

1. Patents;
2. Application of ultrasonic treatment to oleogels;
3. Application of ultrasonic treatment to emulsion systems containing oleogels (emulsions, Pickering emulsions, nanoemulsions, bigels).

### 4.1. Patents

A patent search for inventions addressing the use of ultrasonication in the production of oleogels revealed a wide variety of practical developments in the field of medicine and food technology. The analysis of patents revealed that ultrasonic treatment is applicable to various types of oleogels. Both multicomponent systems (CN107296260A, US20210127720A1, CN112263496A, CN 114617170A) and mono- and bi-components—lecithins (WO2011157428A2), urea derivatives (JP2011184407A) tetrazol and amido pyridyl imidazoles (CN102924397A), carbohydrate derivatives (CN108350012B), 12-hydroxyoctadecanoic acid (CN112980548A), vegetable and animal proteins (CN112617200A), rice bran protein (CN113261594A), whey protein isolate and carboxymethyl chitosan (CN114586969A) are used as gelators. The solvents used in oleogels are mostly edible oils, but synthetic compounds such as organofluorine compounds, individual triglycerides, and hydrocarbons are also used.

The general purpose of ultrasonic treatment is to form a finer and/or more ordered gel structure. For this purpose, the oleogel sample is usually treated at the final stage—during cooling. In some cases, ultrasonic treatment is carried out until the oleogel cools completely, and in other cases it is carried out for a short time only, to form the centers of crystallization. For example, the CN102924397A patent provides a short-term ultrasonic treatment (CN102924397A) of up to 2 min, while the JP2011184407A patent provides a long ultrasonic treatment (1 h to 12 h) while cooling and crystallizing the gel. Depending on the type of oleogel and the expected result, the duration of ultrasonic treatment can vary significantly and can range from 2–3 min to 12 h.

Another application of ultrasound in the designated patents is a solubilization and more uniform distribution of poorly soluble substances in bigels and emulsion gels. In particular, patent CN112980548A uses ultrasound to distribute 12-hydroxyoctadecanoic acid. In patent CN112263496A, ultrasound was used for the same purpose for the surfactant before gelation. In patents CN112617200A, CN113261594A, CN114586969A, ultrasound was used to pre-distribute proteins and carbohydrates in solution. In one of the patents (CN107296260A), ultrasound was used as one possible method of emulsifying the emulsion gel. The duration of treatment in such a case is either not specified (i.e., treatment is required until complete dissolution, regardless of time) or varies from 3 to 30 min.

### 4.2. Application of Ultrasonic Treatment in Oleogels

The method of sonocrystallization significantly influences the properties of the crystalline products, in particular the crystallization process of fats, modifying their initial characteristics. It was necessary to study the component composition of oleogels, processing conditions, and the methods of investigation applied to reveal the characteristics of the influence of ultrasound treatment on the properties of oleogels. The generalized results of the analysis of the ultrasonic treatment application for the oleogels are presented in Table 1.

According to generalized data (Table 1) rapeseed oil [85,86,90,91,93], high-olein sunflower oil [73,77,79], etc., as well as combinations of different oils [50,89,90] are used as oil medium of oleogels, and as gelators—monoglycerides [73,77,79,85,86,91], waxes (candelilla [77,89,90,93], sorghum [94], beeswax [50]), hydrogenated rapeseed oil in combination with MG and/or with lecithin [86,91] and others. To modify the properties of oleogels by ultrasound, both constant exposure [85,86,87,88,90,91,92,93,94] and pulsed exposure are used [50,73,79,89,91]. In this case, the duration of ultrasonic treatment varies from 10 sec to 2 min, or until the sample has been completely crystallized, at a frequency from 100 Hz to 4 MHz.

It is possible to distinguish the commonly studied parameters in the evaluation of the influence of ultrasonic treatment on the properties of the obtained oleogels: morphology and crystal size, by optical, polarization, and scanning electron microscopy methods [73,77,79,86,87,89,91,93,94], oil-binding capacity [73,79,86,88,89,90,91,94], rheological and textural characteristics [73,77,86,87,88,89,91,94], and melting profile [73,77,79,86,87,90,91,94]. Although all fat and fat-containing food systems are susceptible to oxidative processes, there are fewer approaches for evaluating their oxidative stability in studies of oleogels obtained using ultrasound treatment. In isolated studies, the oxidative stability of modified oleogels was assessed using an accelerated method on the Rancimat apparatus [88], as well as by changes in standard peroxide value [90,92,94], acid value [90], thiobarbituric acid values [50,92], and p-Anisidine value [94]. Studies of the possibility of improving the encapsulation of biologically active substances by ultrasonic treatment are also private. For example, oleogel was obtained by ultrasonic treatment based on a mixture of oils and candelilla wax with β-carotene [89].

Currently, there is information about the development of food products based on oleogels subjected to ultrasound treatment, namely cake [29], cookies [88], pate [50], and vegan cream [90]. It is worth noting that most studies have studied the use of ultrasound-treated oleogels in food products but not compared with products based on oleogels without treatment, which makes it difficult to talk about the effect of ultrasound on changing the properties of products. In [50], pates with complete or partial replacement of fat with ethylcellulose or wax oleogels were developed. It was shown that the products obtained had an optimal profile of fatty acids (high PUFA/SFA ratio and low n−6/n−3 ratio) and satisfactory technological and physicochemical properties. The evaluation of sensory properties of samples with waxy oleogel in the composition did not reveal any significant effect on any of the evaluated organoleptic parameters. In contrast, oleogel structured with ethyl cellulose had a negative effect both on sensory properties and resistance to oxidation. In [29], the authors showed the possibility of using oleogels in cake technology. It was determined that these systems can be used in the composition of cake as a substitute for shortening, which will allow for the production of cake rich in unsaturated fatty acids and at the same time having low caloric content, which may be important for the acceptability of the developed product for consumers. One study [90] showed the possibility of developing a stable vegan cream with the addition of oleogel as a fat base, while having physical properties similar to the cream obtained using commercial fats. Although in studies of food products containing ultrasound-treated oleogels there was no control product (comparison product) based on untreated oleogel, the authors [29,50,90] associated the obtained effects, particularly with the use of ultrasound in the development of oleogels. The authors [88] demonstrated the prospect of using ultrasonically processed oleogels in cookie technology. Organoleptic evaluation and general acceptability of such cookies proved to be higher in terms of texture, mouth feel, crispness, and hardness. It was shown that the developed cookies retained their textural properties longer than the butter-based cookies examined as a control sample. In contrast, the unstructured butter-based cookies were too hard and had low acceptability from the beginning of storage. Overall, the authors argue that ultrasound is an effective way to produce strong and stable oleogels, which can be applied in practice as an alternative to solid fats containing saturated and *trans*-isomer fatty acids used in various types of food products. However, a significant disadvantage of studies of food products containing ultrasound-treated oleogels is the lack of untreated samples. This does not allow us to evaluate the contribution of ultrasound treatment on the change of food properties, or to assess the risks that may arise in the process of obtaining food products associated with the distinctive features of the technological process. The possibility of using not only oleogels but also emulsion oleogels in cake technology is shown in a study [29]. The authors determined that these systems, along with oleogels, can be used in a cake as a substitute for shortening.

There are hypotheses about the relationship of microstructural characteristics (morphology and crystal size) with various properties of oleogels, in particular with oil-binding capacity, textural and rheological properties [17,22,95,96]. Oleogels with small crystals form structures with higher oil-binding capacity, hardness, and rheological characteristics. This pattern holds within the limits of studying the effect of ultrasound on a given sample of oleogel with a particular composition of the dispersion medium and the dispersed phase.

#### 4.2.1. Macro- and Microstructure

The analytical review revealed that in most cases, the ultrasonic treatment of oleogels leads to a decrease in particle/crystal size [73,77,79,91,94], but the opposite effect was observed in the article [91], which may be related to the fact that this study studied systems with several gel-formers and different conditions of ultrasonic treatment. When co-crystallizing in binary systems, some gelling agents (e.g., the combined use of monoglycerides and hydrogenated rapeseed oil) are not able to crystallize completely together, forming different crystals or small clusters of crystals [91]. It should be taken into account that the size of the crystals formed is affected by the rate of cooling after ultrasonic treatment. Slow cooling may contribute to the formation of larger crystals, and also during the crystallization of samples with high crystallization kinetics., Due to the rapid crystallization process, ultrasonic treatment may not change this process [73]. Since ultrasonic treatment, by destroying the growing crystals, promotes secondary crystallization, an important factor is the stage of the crystallization process at which the ultrasonic treatment occurs [73,79]. In this case, it is more effective to perform the ultrasonic treatment to influence the crystallization process at the initial stages of crystallization. Low crystallinity ultrasonic treatment can lead to the melting of the resulting crystals, which will reduce the effect of ultrasonic treatment to a minimum [73,79].

The oil-binding capacity of oleogels is an important technological property. As shown previously, ultrasonic treatment can reduce the size of the crystals formed, which in turn can increase the oil-binding capacity [73,79,88,89,90,91,94,97]. This can be explained through a mechanism typical for solid triglyceride mixtures [98]. The authors have shown that triglycerides retain oil more efficiently with smaller particle sizes and more uniform particle distribution, which is due to the mechanism of oil migration. Oil migration in the crystal structure can take place in two ways: Fick’s diffusion and the capillary flow of oil in a three-dimensional network of crystals. The same mechanism may account for the oil-binding capacity of oleogels. Within this concept, the increase in oil-binding capacity with decreasing crystal size may be due to increased capillary forces holding oil in the oleogel structure. It also explains the negative effect on oil-binding capacity noted in [91], in which there is an increase in particle size and a decrease in oil-binding capacity. The insignificant changes in the oil-binding capacity of oleogels during treatment with ultrasound in a standing wave [85] are because, during the formation of ribbon-like structures described by the authors, there is an increase in the diffusion flow of oil, but the intensity of capillary flow does not increase. In [77], the authors studied different systems that responded differently to ultrasonic treatment. It was shown that the decrease in crystal size as a result of ultrasonic treatment was not enough to change the physical properties of oleogel, except for the sample with the longest treatment duration and amplitude. In [73], a synergistic effect between low cooling rate and ultrasonic treatment was revealed, allowing to oleogel to be obtained with an improved oil binding capacity and a reduced amount of gelator, without changing the physical properties. Similar results were obtained in [97]; however, the authors showed that the relationship between the reduction of particle size and increase of oil-binding capacity is maintained at ultrasonic treatment up to 450 W. At higher power, larger particles were formed and the oil-binding capacity decreased.

#### 4.2.2. Rheological and Texture Properties

Special attention is paid to the study of rheological and textural characteristics in the development of oleogels. It was shown that in most cases exposure to ultrasound leads to an increase in the rheological properties (G’, G’’) [73,79,88,89,91,94], but there are examples where these parameters decreased [77,91]. Similar changes are shown for the hardness index. For example, in [73,77,79,88,89,91,94], an increase in hardness is revealed, while in [77,79,85,91], insignificant changes in elasticity or hardness of oleogels under ultrasonic exposure, despite the reduction of crystal size, are shown. It has been noted that ultrasonic treatment results in a more elastic crystal mesh [73], but ultrasonic treatment may not change G’ during rapid cooling. The increase in hardness of oleogels can be explained by the formation under ultrasonic treatment of smaller crystals having a larger contact area, which contributes to a harder structure. In [73], it was noted that a high cooling rate can increase the hardness of ultrasound-treated MG oleogels, but this effect is found only at low concentrations of MG (3%); at higher concentrations (up to 6%) no significant changes were found. Studies of changes in adhesion under ultrasound exposure revealed both an increase [73,79] and a decrease [79] in this index, which is associated with different concentrations of the gel-forming agent. When determining the cohesion, it is shown that this index may not change [73,79], or decrease [79]. Some studies have also evaluated the effect of ultrasonic treatment on stickiness, elastic modulus, and yield strength, and found an increase in these indicators [85,88,99]. It should be taken into account that not all oleogels harden with decreasing crystal particle size [100]. In this case, ultrasonic treatment can potentially reduce the hardness of the oleogel. In general, the results of rheological and textural properties are consistent with the results of oil-binding capacity (softer oleogels had greater oil loss).

#### 4.2.3. Thermal Properties

When evaluating the melting profile, a number of papers [73,77] noted that despite the lack of influence of ultrasonic treatment on the solid fat content, the melting enthalpy and melting onset temperature for some treated oleogel samples slightly decreased, which may indicate the formation of a weaker crystal network. Even though the decrease in crystal size may indicate a stronger interaction between the crystals, which in turn may lead to a narrow melting profile, this effect is not always observed [77]. However, the change in the indices characterizing the melting process may depend on the cooling rate of the oleogels after ultrasonic treatment, since slower cooling can promote the inclusion of compounds with lower melting temperatures (e.g., compounds present in commercial MG or the oil phase) into the crystal network [73]. In [79], the possible change of melting profile was also shown, in particular, the melting onset temperature may increase with increasing concentration of the gel-forming agent. At the same time, the authors noted that there are no reliable differences in the melting temperatures associated with the effect of ultrasonic treatment. Although in the study [91] ultrasound treatment, in general, did not affect the change in the phase diagram of oleogels, a sample was found in which there was an increase in the melting peak, which had shifted closer to the phase transitions characteristic of gelators. The authors propose the use of ultrasonic treatment as a tool to reduce incompatibility among gelators and improve the formation of a more packed crystal network in binary oleogels. In general, the absence of changes in the oleogels may indicate that there is no change in the chemical composition of the substances responsible for gelation in these systems, since these are inherent in the peaks observed in the thermograms. A study [94] noted a decrease in the enthalpy of melting in samples treated with ultrasound. The authors suggest that ultrasound treatment narrows the melting range both by increasing the initial melting temperature and by reducing the final melting temperature. 

#### 4.2.4. Oxidative Stability

The oxidative stability of food systems containing fats is an important hygienic indicator. Oleogels are systems containing large amounts of oils (over 90%), which are subject to oxidative processes. It is known that the oxidative stability of oleogels may depend on the component composition of the gelling agent or their mixture [21,101]. During ultrasonic treatment, the temperature of the samples increases due to cavitation [77]. Depending on the duration and power of ultrasonic treatment, the temperature of fat systems, including oleogels [73,77], increases, and an unpleasant odor may appear, but primary oxidation products may not be formed [102]. The occurrence of an unpleasant odor may limit the use of products, so it is important to select a processing mode that changes the properties but does not produce an unpleasant odor and primary oxidation products are not formed. An analytical review of publications revealed a small number of studies on the oxidative stability of oleogels obtained by ultrasonic treatment. In [88], it was shown that ultrasonically treated oleogels have better oxidative stability than untreated samples. Presumably, this is due to changes as a result of cavitation during ultrasonic treatment, which leads to an increase in the number of nucleation centers, and the formation of hydrogen bonds, resulting in a stronger three-dimensional network and less oil separation and consequently slowing down oxidation processes. The formation of hydrogen bonds in ultrasound-treated systems is confirmed by the results of FTIR analysis [97]. However, according to the data presented in the literature, a full analysis of the factors influencing changes in the oxidative stability of oleogels as a result of ultrasonic treatment is not possible at present. This is due to the small number of studies, the choice of different indicators (PV, TBARS, p-AnV) and methods (Rancimat, NMR) that characterize the oxidative stability, and the choice of the reference sample (using oil (oil mixture) as a comparison sample, rather than oleogel without ultrasonic treatment). For example, work [90] showed that oleogel prepared with ultrasound treatment had no reliable differences from the original oil in terms of peroxide number immediately after preparation, but after 30 days of storage had a lower index, indicating better oxidative stability. However, the absence of a control sample of oleogel without ultrasound treatment does not allow us to argue that the improvement in the oxidative stability of oleogels compared with the original oil is due to ultrasound exposure.

#### 4.2.5. Miscellaneous

Currently, oleogels are studied not only as an alternative to solid fats, but also as carriers of biologically active substances [5]. The application of ultrasonic treatment to oleogels containing biologically active additives can increase the preservation of biologically active substances [89]. In [89], it was shown that ultrasound treatment of oleogels enriched with β-carotene resulted in the slower degradation of β-carotene compared to untreated oleogels. The authors suggest that this is because ultrasound treatment promotes crystal aggregation, which leads to denser crystal networks and improves carotene binding. In an in vitro model, ultrasound treatment of oleogels has been shown to reduce β-carotene release in the intestinal digestion phase.

In a number of studies [73,89,91,93,94,97], X-ray analysis was performed to investigate the effect of ultrasonic treatment on the properties of oleogels in more detail. The analysis revealed no change in crystal polymorphism. The study [97] showed that, with increasing ultrasound power, the intensity of the diffraction peak increased and the crystallinity of the samples increased, while there was no change in the crystal shape.

### 4.3. Application of Ultrasonic Treatment to Emulsion Systems Containing Oleogels

Along with the study of the effect of ultrasonic treatment on the properties of oleogels, part of the research is devoted to the use of ultrasonic treatment to produce emulsion systems containing oleogels (Table 2).

Among the studies on the preparation of emulsions containing oleogels (Table 2), the following substances were used as surfactants, emulsifiers, and stabilizers: monoglycerides [77,103,106,113,116], tween 80 [106,113], lecithin [103,108], k-carrageenan [99,112], nanocrystalline cellulose [109,110,114], whey protein concentrate [99], cream [90], soy protein isolate [97], sodium caseinate [108], insoluble tea protein [112], fish collagen [104], Pluronic F-68 [106], chitosan [107], tween 20 [113], sorbitan monooleate [108], lupeol [113], lactoferrin [105], phytosterols, tea saponin, *Quillaja* saponin, and octenyl succinate starch [108]. The following oils were used in the oil phase for obtaining the emulsion: sunflower [115], including high-oleic [29,77,111], corn [104,105,107], olive [111], including extra virgin [106,110], rapeseed [90,99], soybean [109,114], cotton [29] canola oil [113], coconut oil [113], fish oil [112], walnut oil [97], refined palm oil [90], linseed oil [90], as well as Industrial blend oil [116] and fat blend [111]. The following components were used to structure emulsion-based oleogels: carnauba wax [29,104,111,116], rice bran wax [97], candelilla wax [77,90,105], beeswax [109,114], behenyl alcohol [104,105], milk fat [90], solid fat [103], fully hydrogenated Crambe oil [77], ethyl cellulose [107]. β-carotene [29,111,114,115,116], curcumin [105,106,113], vitamin D_3_ [29,116] vitamin A [29,116], quercetin [113], astaxanthin [104] were used as bioactive substances to deliver or study their preservation.

The procedure for ultrasonic treatment of emulsion systems containing oleogels is described in various articles with varying degrees of detail. Nevertheless, it seems possible to specify some characteristics of the treatment. In particular, the maximum treatment power is 500 W, the frequency is 26 kHz, and the treatment duration is 90 min. Both pulse and constant treatment modes are used. Ultrasonic treatment is used for all types of oleogels (polymeric, crystalline, and self-assembled) based on various oils. The main purpose of using ultrasonic treatment in these types of products is homogenization. In addition, ultrasound is used to create the desired structure during cooling and for the polymerization of structure-forming agents.

In addition to studies of the structure and properties of anhydrous oleogels, the authors of the emulsion system studies also evaluated the stability of emulsions and the release of biologically active substances. The main method for determining the stability of emulsions in the above studies was the visual determination of emulsion delamination, and creaming, as well as a study of the droplet size distribution. To study bioavailability, in vitro methods were mainly used to break emulsions under different conditions. One study [113] used an in vivo cell culture model to evaluate the bioavailability of the substances from the emulsion.

#### 4.3.1. Micro- and Macrostructure

The study of micro- and macrostructural characteristics is an important component in the development of emulsion gels, in particular, to evaluate the application of ultrasonic exposure. In the study [104], ultrasonic treatment was applied to obtain nanoemulsions with smaller particle sizes, which can be used for the delivery of biologically active substances. In [106], the reduction of emulsion particle size due to ultrasonic treatment is shown. However, ultrasonic treatment does not always lead to a decrease in crystal size in emulsion systems. For example, it was noted in [77] that at 5% and 25% water content, crystal size in samples obtained with ultrasonic treatment did not change.

In the study of oil-binding ability, the authors [99] showed that ultrasonic treatment led to an increase in this parameter in bigels. A similar pattern was found for emulsion oleogel samples as a result of ultrasound exposure in [103]. In general, the treatment of emulsion oleogels with ultrasound leads to a decrease in particle size and an increase in oil-binding capacity.

#### 4.3.2. Rheological and Texture Properties

In the use of ultrasound treatment in the production of emulsions in [103], it was shown that ultrasound-treated MG:HF emulsions formed more organized and stable water droplets at 30 W. Positive effects of ultrasonic treatment have also been shown for LE and HF:LE emulsions at 30 W power, resulting in improved hardness, modulus of elasticity and as previously shown oil-binding capacity. In addition, in [99], it was shown that a decrease in the gel particle size as a result of ultrasound treatment led to an increase in the rheological and textural properties of bigels (MAG+k-carrageenan), and similar results were obtained for emulsion olegels (MAG+candelilla wax+hardfat) [77]. In general, the results of these studies indicate that the use of ultrasonic treatment in emulsion gels contributes to an increase in the rheological and textural characteristics due to the reduction of particle size and greater homogeneity of their distribution.

#### 4.3.3. Biologically Active Substances

In [113], it was shown that the bioavailability of bioactive substances contained in emulsion-based oleogels may depend on the type of bioactive substances. It was shown that the bioavailability of lupeol contained in emulsion oleogel increased, while the bioavailability of curcumin and quercitin did not change. The study in [106] showed that bigels, which are characterized by smaller particle sizes, are an effective matrix for curcumin encapsulation. At the same time, it was determined that the capture efficiency of curcumin in the bigel is 60% higher than in the oil particles without a gelling agent. The effect of ultrasonic treatment on emulsion oleogels with biologically active substances may lead to increased the safety and efficiency of encapsulation of these biologically active substances [108,113,114,115]. In part of [104,105,106,114], ultrasonic treatment was used as a stage to produce emulsion oleogels containing biologically active substances. However, the absence of a control sample obtained without ultrasonic treatment in these studies does not allow us to evaluate the contribution of ultrasonic treatment on the change in the properties of emulsion oleogels and the bioavailability/encapsulation of biologically active substances.

## 5. Conclusions

The increasing demand for high-quality, safe food products that meet the body’s needs is driving an ongoing search for ingredients, methods, and approaches to obtain products with improved properties. Oleogels may become a viable alternative to saturated and *trans*-isomer fatty acids in foods in the future. Strategies in the field of oleogels are directed to studying the factors and methods of influence on various (physicochemical, organoleptic, physiological) properties of oleogels and ultimately aim to obtain finished products with a modified lipid component which satisfies consumer demands. 

This review presents an analysis of publications devoted to the use of ultrasonic treatment in obtaining oleogels (or emulsion oleogels), as well as evaluating the effect of ultrasound on the properties of oleogels. 

Based on the analysis, it is shown that ultrasound treatment of oleogels leads to a reduction of crystal/particle size of oleogels, and an increase in oil-binding capacity, hardness, and rheological characteristics. The main factors influencing the efficiency of ultrasonic treatment of oleogels and emulsion systems containing oleogels were distinguished: the cooling rate of oleogels after ultrasonic treatment; the crystallization of oleogels during ultrasonic treatment; the composition of oleogels, including minor components; nature and concentration of gelling agents; treatment duration and amplitude (power).

It was found that few studies accurately indicate the frequency, power, and duration of ultrasound treatment. Although the general changes in the properties of oleogels, including emulsion oleogels, under the influence of ultrasound treatment, are the reduction in crystal size, an increase of oil-binding ability, hardness, and rheological characteristics, there are studies in which no changes or the opposite effect was observed. 

Modification of the properties of oleogels, including ultrasonic treatment, is a promising direction for obtaining new lipid systems providing necessary technological and sensory properties of finished products.

## Figures and Tables

**Table 1:** Application of ultrasonic treatment to produce oleogels.

No	Gel Composition	Processing Options	Applications	References
1	Rapeseed oil, monoglyceride (MG)	- Ultrasonic standing waves; - Treatment before crystallization of the sample, cooling rate 3.8 °C/min and 1.3 °C/min; - Resonant frequency 2.25 MHz	To decrease effective stiffness; For use in the food, pharmaceutical, and cosmetic industries.	[85]
2	Fully hydrogenated rapeseed oil, rapeseed oil, MG, lecithin (LE)	- At a temperature of 55 °C; - Duration 10 s; - 20 kHz, amplitude 50% (50 ± 2 W, 1 W/mL)	To increase hardness; For use in food.	[86]
3	Glyceryl tridecanoate, *α*-cellulose particles, sorbitan monostearate (Span 60), sorbitan monopalmitate (Span 40)	- Probe diameter 0.6 mm; - Duration 2 min; - Frequency 40 kHz, power 480 W	Potential application in food industry.	[87]
4	Medium chain triglycerides, ethyl cellulose	- Probe diameter 20 mm; - Frequency 20 kHz, power 240 W, duty cycle 10%; - At 40 °C	To increase OBC, rheology, texture and oxidative stability; For use in bakery products (cookies).	[88]
5	Peanut oil, pine nut oil, walnut oil, candelilla wax, *β*-carotene	- Probe diameter 6.36 mm; - 20 kHz at 95 W (amplitude 10%), ultrasound intensity 0.47 ± 0.03 W/mL; - Duration 20 s (2 s on, 2 s off, actual time 10 s)	To increase rheology, texture and OBC of oleogels; To improve the protection of β-carotene; To reduce the β-carotene released during intestinal digestion; For use in food and pharmaceutical industries.	[89]
6	Rapeseed oil, linseed oil, candelilla wax	- 26 kHz, 72 W, amplitude 100%, impulse 100%; - Duration 15 s; - Sonotrode immersion 15 mm	For use in vegan creams.	[90]
7	Fully hydrogenated rapeseed oil, rapeseed oil, MG	- Probe diameter 12.7 mm; - 20 kHz, amplitude 50% (56 W); - Duration 10 s, 30 s or 10 s (10 s on/10 s off)	To increase hardness and OBC; For application in food industry.	[91]
8	Soybean oil, peanut oil, acidic blueberry powder, alkaline blueberry powder, resveratrol, sitosterol, LE	- Processing time 1 min	For application in food industry.	[92]
9	Rapeseed oil, sunflower wax, candelilla wax, MG	- Ultrasonic standing waves; - Voltage 25 V, 10 V and 5 V for cameras 1 MHz, 2 MHz and 4 MHz, respectively; - Ultrasonic treatment until complete crystallization	For use in cosmetics, pharmaceutical, chemical, and food industries.	[93]
10	High oleic sunflower oil, MG	- Probe diameter 3.2 mm; - 20 kHz, 96 W; - 30 s—3 pulses: 10 s on/5 s off; - Cooling rate 0.1 and 10 °C/min	To reduce crystal size; To increase rheology, texture and OBC of oleogels; For application in food industry.	[73]
11	Fish oil, sorghum wax	- Power 110 W, frequency 40 kHz; - At 85 °C; - Duration 5 min	To reduce oil loss by reducing the crystal size. For application in food industry.	[94]
12	High-olein sunflower oil, MG	- Probe diameter 3.2 mm; - 20 kHz, power 96 W, 30 s in pulse mode: 10 s on/5 s off; - Cooling rate 0.1 and 10 °C/min	To increase OBC and hardness. For use as fat substitutes in bakery.	[79]
13	Olive oil, linseed oil, fish oil, ethyl cellulose, beeswax, sorbitan monostearate	- 100 Hz; - 2 cycles 1 min on/30 s off	For use as animal fat replacers for pork liver pâtés.	[50]
14	High-olein sunflower oil, fully hydrogenated crambe oil, monoacylglycerols (MAG), candelilla wax	- Probe diameter 3.2 mm or 12.7 mm - 20 kHz, vibration amplitude 120–216 µm or 60–108 µm	To increase the hardness. For application in food industry.	[77]

**Table 2:** Application of ultrasonic treatment for obtaining emulsion systems containing oleogels.

No	Emulsion Composition	Processing Options	Applications	References
1	Walnut oil, rice bran wax, soy protein isolate, phosphatidylserine, water	- Submersible titanium probe—6 mm; - Processing time 15 min; - Ultrasonic power: 150, 300, 450 and 600 W	To improve the oxidation stability and surface characteristics; For use in oils with a high unsaturated fatty acid.	[97]
2	Hardfat (HF), MG, LE, water	- Not described	To improve hardness, elastic modulus and OBC; For use in food industry.	[103]
3	Corn oil, carnauba wax, behenyl alcohol, astaxanthin, fish collagen, water	- Pressure amplitude 70%; - Duration 10 min in pulse mode 4 s on/6 s off	For use as a nutraceutical delivery system.	[104]
4	Corn oil, carnauba wax, behenyl alcohol, lactoferrin, transglutaminase, curcumin, water	- 20 kHz; - 3 min in pulse mode 4 s on/6 s off	For use as a nutraceutical delivery system.	[105]
5	Canola oil, MAG, kappa-carrageenan, whey protein concentrate, potassium chloride, water	- 20 kHz, amplitude 50%, (specific energy density 72 J/mL) for a sample volume of 100 mL; - duration 2 min	For use as a nutraceutical delivery system.	[99]
6	Extra virgin olive oil, MG, curcumin, Pluronic F-68, Tween-80, water	- 40% duty cycle load and given output power level and time	For use as a nutraceutical delivery system.	[106]
7	Corn oil, ethyl cellulose, chitosan, deionized water	- 500 W; - Duration 10 min (3 s on, 3 s off)	For use as a nutraceutical delivery system.	[107]
8	Flavor oils, phytosterols, tea saponin, *Quillaja* saponin, octenylsuccinate starch, sodium caseinate, sorbitan monooleate, soybean lecithin, water	- Horn tip 95 mm; - 20 kHz, 200–400 W; - Duration 5 s on, 5 s off	For use as a nutraceutical delivery system.	[108]
9	Milk fat, refined palmoil, rapeseed oil, linseed oil, candelilla wax, dairy cream, soy drink, water	- 26 kHz, 48 ± 2 W, energy 2.68 ± 0.003 kJ, speed 18.4 J/mL, amplitude 80%, impulse 100%; - Sonotrode immersion 15 mm, - Duration 1 min	For use in vegan creams.	[90]
10	Soybean oil, sodium chloride, beeswax, cellulose nanocrystals, deionized water	- Pressure amplitude 60%; - Duration 2 min (3 s on/3 s off)	For use in emulsions for food applications	[109]
11	Extra virgin olive oil, hexadecane, casein sodium salt, cellulose nanocrystal, calcium chloride, sodium chloride, fluorescein sodium salt, Milli-Q water	- 400 W at 60% power; - within three cycles lasting 1 min	For use as a nutraceutical delivery system.	[110]
12	High oleic sunflower oil, hazelnut oil, olive oil, blend fat, carnauba wax, *β*-carotene, water	- 200 W, amplitude 100%; - Duration 10 min	For use in food production.	[111]
13	Fish oil, tea water-insoluble protein, κ-carrageenan, deionized water	- Ultrasound power was 384 W; - Duration 4 min (5 s on/5 s off)	For use in food production.	[112]
14	Canola oil, coconut oil, curcumin, quercetin, lupeol, Tween 20 (Polyoxyethylene-20-sorbitan monolaurate), Tween 80 (Polyoxyethylene-20-sorbitan monooleate), MG, water	- Ultrasonic bath for 30 min, ultrasonic homogenizer 200 W and amplitude 90%	For use in food production.	[113]
15	Soybean oil, beeswax, β-carotene, cellulose nanocrystals, sodium chloride, hydrogen chloride, sodium hydroxide, water	- Pressure amplitude 60%; - Duration 2 min (3 s on/3 s off)	For use as a nutraceutical delivery system.	[114]
16	Sunflower oil, sanxan, *β*-carotene, sodium chloride, calcium chloride, water	- Duration 20 min; - At 30 °C	For use as a nutraceutical delivery system.	[115]
17	High-oleic sunflower oil, candelilla wax, MAG, fully hydrogenated crambe oil, water	- Probe diameter 3.2 mm; - 20 kHz, amplitude 216 μm; - Duration 3 min	To increase the hardness. For application in the food industry.	[77]
18	Shortening, cotton oil, high-oleic sunflower oil, carnauba wax, citric acid, potassium sorbate, salt, emulsifiers, *β*-carotene, vitamin A, and D_3_, water	- 100% amplitude; - Duration 3 min - Secondary processing: - 100% amplitude; - Duration 2 min	For use in batter.	[29]
19	Industrial blend oil, carnauba wax, high-oleic sunflower oil, sunflower lecithin, MG, β-carotene, vitamin A, vitamin D_3_, citric acid, potassium sorbate, sodium chloride, skim milk, water	- 100% amplitude; - Duration 10 min	For use in food production.	[116]

## Data Availability

Not applicable.

## References

[1] Jadhav H.B., Annapure U.S. (2022). Triglycerides of medium-chain fatty acids: A concise review. J. Food Sci. Technol..

[2] Temkov M., Mureșan V. (2021). Tailoring the structure of lipids, oleogels and fat replacers by different approaches for solving the trans-fat issue—A review. Foods.

[3] Shahidi F., Hossain A. (2022). Role of Lipids in Food Flavor Generation. Molecules.

[4] Bhandari S.D., Delmonte P., Honigfort M., Yan W., Dionisi F., Fleith M., Iassonova D., Bergeson L.L. (2020). Regulatory changes affecting the production and use of fats and oils: Focus on partially hydrogenated oils. J. Am. Oil Chem. Soc..

[5] Frolova Y.V., Kochetkova A.A., Sobolev R.V., Vorobyeva V.M., Kodentsova V.M. (2021). Oleogels as prospective nutritional ingredients of lipid nature. Nutr. Issue.

[6] Patel A.R., Nicholson R.A., Marangoni A.G. (2020). Applications of fat mimetics for the replacement of saturated and hydrogenated fat in food products. Curr. Opin. Food Sci..

[7] Li L., Liu G., Bogojevic O., Pedersen J.N., Guo Z. (2022). Edible oleogels as solid fat alternatives: Composition and oleogelation mechanism implications. Compr. Rev. Food Sci. Food Saf..

[8] Pinto T.C., Martins A.J., Pastrana L., Pereira M.C., Cerqueira M.A. (2021). Oleogel-based systems for the delivery of bioactive compounds in foods. Gels.

[9] Feichtinger A., Scholten E. (2020). Preparation of protein oleogels: Effect on structure and functionality. Foods.

[10] Pușcaș A., Mureșan V., Muste S. (2021). Application of analytical methods for the comprehensive analysis of oleogels—A review. Polymers.

[11] Wang Z., Chandrapala J., Truong T., Farahnaky A. (2022). Oleogels prepared with low molecular weight gelators: Texture, rheology and sensory properties, a review. Crit. Rev. Food Sci. Nutr..

[12] Hwang H.-S. (2020). A critical review on structures, health effects, oxidative stability, and sensory properties of oleogels. Biocatal. Agric. Biotechnol..

[13] Martins A.J., Vicente A.A., Cunha R.L., Cerqueira M.A. (2018). Edible oleogels: An opportunity for fat replacement in foods. Food Funct..

[14] Co E.D., Marangoni A.G. (2012). Organogels: An alternative edible oil-structuring method. J. Am. Oil Chem. Soc..

[15] Bascuas S., Hernando I., Moraga G., Quiles A. (2020). Structure and stability of edible oleogels prepared with different unsaturated oils and hydrocolloids. Int. J. Food Sci. Technol..

[16] Pang M., Shi Z., Lei Z., Ge Y., Jiang S., Cao L. (2020). Structure and thermal properties of beeswax-based oleogels with different types of vegetable oil. Grasas Y Aceites.

[17] Frolova Y., Sarkisyan V., Sobolev R., Makarenko M., Semin M., Kochetkova A. (2022). The Influence of Edible Oils’ Composition on the Properties of Beeswax-Based Oleogels. Gels.

[18] Dassanayake L.S.K., Kodali D.R., Ueno S. (2011). Formation of oleogels based on edible lipid materials. Curr. Opin. Colloid Interface Sci..

[19] Lim J., Hwang H.-S., Lee S. (2017). Oil-structuring characterization of natural waxes in canola oil oleogels: Rheological, thermal, and oxidative properties. Appl. Biol. Chem..

[20] Frolova Y., Sobolev R., Kochetkova A. (2021). Influence of oil combinations on the structural properties of oleogels. E3S Web Conf..

[21] Hwang H.S., Fhaner M., Winkler-Moser J.K., Liu S.X. (2018). Oxidation of fish oil oleogels formed by natural waxes in comparison with bulk oil. Eur. J. Lipid Sci. Technol..

[22] Palla C., de Vicente J., Carrín M.E., Ruiz M.J.G. (2019). Effects of cooling temperature profiles on the monoglycerides oleogel properties: A rheo-microscopy study. Food Res. Int..

[23] Martins A.J., Cerqueira M.A., Pastrana L.M., Cunha R.L., Vicente A.A. (2019). Sterol-based oleogels’ characterization envisioning food applications. J. Sci. Food Agric..

[24] Sarkisyan V., Sobolev R., Frolova Y., Malinkin A., Makarenko M., Kochetkova A. (2021). Beeswax fractions used as potential oil gelling agents. J. Am. Oil Chem. Soc..

[25] Gravelle A.J., Barbut S., Marangoni A.G. (2012). Ethylcellulose oleogels: Manufacturing considerations and effects of oil oxidation. Food Res. Int..

[26] Espert M., Salvador A., Sanz T. (2020). Cellulose ether oleogels obtained by emulsion-templated approach without additional thickeners. Food Hydrocoll..

[27] Oh I.K., Amoah C., Lim J., Jeong S., Lee S. (2017). Assessing the effectiveness of wax-based sunflower oil oleogels in cakes as a shortening replacer. LWT.

[28] Giacomozzi A.S., Carrín M.E., Palla C.A. (2018). Muffins elaborated with optimized monoglycerides oleogels: From solid fat replacer obtention to product quality evaluation. J. Food Sci..

[29] Pehlivanoglu H., Özülkü G., Yildirim R.M., Demirci M., Toker O.S., Sağdiç O. (2018). Investigating the usage of unsaturated fatty acid-rich and low-calorie oleogels as a shortening mimetics in cake. J. Food Process. Preserv..

[30] Oh I.K., Lee S. (2018). Utilization of foam structured hydroxypropyl methylcellulose for oleogels and their application as a solid fat replacer in muffins. Food Hydrocoll..

[31] Kim M., Hwang H.-S., Jeong S., Lee S. (2022). Utilization of oleogels with binary oleogelator blends for filling creams low in saturated fat. LWT.

[32] Doan C.D., Patel A.R., Tavernier I., De Clercq N., Van Raemdonck K., Van de Walle D., Delbaere C., Dewettinck K. (2016). The feasibility of wax-based oleogel as a potential co-structurant with palm oil in low-saturated fat confectionery fillings. Eur. J. Lipid Sci. Technol..

[33] Palla C.A., Wasinger M.F., Carrín M.E. (2021). Monoglyceride oleogels as fat replacers in filling creams for sandwich cookies. J. Sci. Food Agric..

[34] Palla C., Giacomozzi A., Genovese D.B., Carrín M.E. (2017). Multi–objective optimization of high oleic sunflower oil and monoglycerides oleogels: Searching for rheological and textural properties similar to margarine. Food Struct..

[35] Hwang H.-S., Singh M., Bakota E.L., Winkler-Moser J.K., Kim S., Liu S.X. (2013). Margarine from organogels of plant wax and soybean oil. J. Am. Oil Chem. Soc..

[36] Hwang H.-S., Singh M., Winkler-Moser J.K., Bakota E.L., Liu S.X. (2014). Preparation of margarines from organogels of sunflower wax and vegetable oils. J. Food Sci..

[37] Rodríguez-Hernández A.K., Pérez-Martínez J.D., Gallegos-Infante J.A., Toro-Vazquez J.F., Ornelas-Paz J.J. (2021). Rheological properties of ethyl cellulose-monoglyceride-candelilla wax oleogel vis-a-vis edible shortenings. Carbohydr. Polym..

[38] Ye X., Li P., Lo Y.M., Fu H., Cao Y. (2019). Development of novel shortenings structured by ethylcellulose oleogels. J. Food Sci..

[39] Pintado T., Cofrades S. (2020). Quality characteristics of healthy dry fermented sausages formulated with a mixture of olive and chia oil structured in oleogel or emulsion gel as animal fat replacer. Foods.

[40] Zampouni K., Soniadis A., Dimakopoulou-Papazoglou D., Moschakis T., Biliaderis C.G., Katsanidis E. (2022). Modified fermented sausages with olive oil oleogel and NaCl–KCl substitution for improved nutritional quality. LWT.

[41] Utrilla M.C., Ruiz A.G., Soriano A. (2014). Effect of partial replacement of pork meat with an olive oil organogel on the physicochemical and sensory quality of dry-ripened venison sausages. Meat Sci..

[42] Kouzounis D., Lazaridou A., Katsanidis E. (2017). Partial replacement of animal fat by oleogels structured with monoglycerides and phytosterols in frankfurter sausages. Meat Sci..

[43] Panagiotopoulou E., Moschakis T., Katsanidis E. (2016). Sunflower oil organogels and organogel-in-water emulsions (part II): Implementation in frankfurter sausages. LWT.

[44] Franco D., Martins A.J., López-Pedrouso M., Purriños L., Cerqueira M.A., Vicente A.A., Pastrana L.M., Zapata C., Lorenzo J.M. (2019). Strategy towards replacing pork backfat with a linseed oleogel in frankfurter sausages and its evaluation on physicochemical, nutritional, and sensory characteristics. Foods.

[45] Wolfer T.L., Acevedo N.C., Prusa K.J., Sebranek J.G., Tarté R. (2018). Replacement of pork fat in frankfurter-type sausages by soybean oil oleogels structured with rice bran wax. Meat Sci..

[46] Barbut S., Wood J., Marangoni A. (2016). Potential use of organogels to replace animal fat in comminuted meat products. Meat Sci..

[47] Barbut S., Tiensa B.E., Marangoni A.G. (2021). Partial fat replacement in liver pâté using canola oil organogel. LWT.

[48] Alejandre M., Astiasarán I., Ansorena D., Barbut S. (2019). Using canola oil hydrogels and organogels to reduce saturated animal fat in meat batters. Food Res. Int..

[49] Martins A.J., Lorenzo J.M., Franco D., Pateiro M., Domínguez R., Munekata P.E.S., Pastrana L.M., Vicente A.A., Cunha R.L., Cerqueira M.A. (2020). Characterization of enriched meat-based pâté manufactured with oleogels as fat substitutes. Gels.

[50] Gómez-Estaca J., Herrero A.M., Herranz B., Álvarez M.D., Jiménez-Colmenero F., Cofrades S. (2019). Characterization of ethyl cellulose and beeswax oleogels and their suitability as fat replacers in healthier lipid pâtés development. Food Hydrocoll..

[51] Moghtadaei M., Soltanizadeh N., Goli S.A.H., Sharifimehr S. (2021). Physicochemical properties of beef burger after partial incorporation of ethylcellulose oleogel instead of animal fat. J. Food Sci. Technol..

[52] Moghtadaei M., Soltanizadeh N., Goli S.A.H. (2018). Production of sesame oil oleogels based on beeswax and application as partial substitutes of animal fat in beef burger. Food Res. Int..

[53] Khiabani A.A., Tabibiazar M., Roufegarinejad L., Hamishehkar H., Alizadeh A. (2020). Preparation and characterization of carnauba wax/adipic acid oleogel: A new reinforced oleogel for application in cake and beef burger. Food Chem..

[54] Adili L., Roufegarinejad L., Tabibiazar M., Hamishehkar H., Alizadeh A. (2020). Development and characterization of reinforced ethyl cellulose based oleogel with adipic acid: Its application in cake and beef burger. LWT.

[55] Özer C.O., Çelegen Ş. (2021). Evaluation of quality and emulsion stability of a fat-reduced beef burger prepared with an olive oil oleogel-based emulsion. J. Food Process. Preserv..

[56] Silva T.J., Fernandes G.D., Bernardinelli O.D., Silva E.C.d.R., Barrera-Arellano D., Ribeiro A.P.B. (2021). Organogels in low-fat and high-fat margarine: A study of physical properties and shelf life. Food Res. Int..

[57] Blake A.I., Marangoni A.G. (2015). Factors affecting the rheological properties of a structured cellular solid used as a fat mimetic. Food Res. Int..

[58] Tirgarian B., Yadegari H., Bagheri A., Neshagaran E., Mardani M., Farmani J. (2023). Reduced-fat chocolate spreads developed by water-in-oleogel emulsions. J. Food Eng..

[59] Pandolsook S., Kupongsak S. (2017). Influence of bleached rice bran wax on the physicochemical properties of organogels and water-in-oil emulsions. J. Food Eng..

[60] Chemat F., Khan M.K. (2011). Applications of ultrasound in food technology: Processing, preservation and extraction. Ultrason. Sonochem..

[61] Gallo M., Ferrara L., Naviglio D. (2018). Application of ultrasound in food science and technology: A perspective. Foods.

[62] Welti-Chanes J., la Peña M.M.-d., Jacobo-Velázquez D.A., Martín-Belloso O. (2017). Opportunities and challenges of ultrasound for food processing: An industry point of view. Ultrasound Adv. Food Process. Preserv..

[63] Vilkhu K., Mawson R., Simons L., Bates D. (2008). Applications and opportunities for ultrasound assisted extraction in the food industry—A review. Innov. Food Sci. Emerg. Technol..

[64] Dai J., Bai M., Li C., Cui H., Lin L. (2020). Advances in the mechanism of different antibacterial strategies based on ultrasound technique for controlling bacterial contamination in food industry. Trends Food Sci. Technol..

[65] Bhargava N., Mor R.S., Kumar K., Sharanagat V.S. (2021). Advances in application of ultrasound in food processing: A review. Ultrason. Sonochem..

[66] Arvanitoyannis I.S., Kotsanopoulos K.V., Savva A.G. (2017). Use of ultrasounds in the food industry–Methods and effects on quality, safety, and organoleptic characteristics of foods: A review. Crit. Rev. Food Sci. Nutr..

[67] Natarajan S., Ponnusamy V. (2020). WITHDRAW: A review on the applications of ultrasound in food processing. Mater. Today Proc..

[68] Chandrapala J. (2015). Low intensity ultrasound applications on food systems. Int. Food Res. J..

[69] Zhou L., Zhang J., Xing L., Zhang W. (2021). Applications and effects of ultrasound assisted emulsification in the production of food emulsions: A review. Trends Food Sci. Technol..

[70] Dzah C.S., Duan Y., Zhang H., Wen C., Zhang J., Chen G., Ma H. (2020). The effects of ultrasound assisted extraction on yield, antioxidant, anticancer and antimicrobial activity of polyphenol extracts: A review. Food Biosci..

[71] Munekata P.E., Alcántara C., Žugčić T., Abdelkebir R., Collado M.C., García-Pérez J.V., Jambrak A.R., Gavahian M., Barba F.J., Lorenzo J.M. (2020). Impact of ultrasound-assisted extraction and solvent composition on bioactive compounds and in vitro biological activities of thyme and rosemary. Food Res. Int..

[72] Huang G., Chen S., Dai C., Sun L., Sun W., Tang Y., Xiong F., He R., Ma H. (2017). Effects of ultrasound on microbial growth and enzyme activity. Ultrason. Sonochem..

[73] Giacomozzi A., Palla C., Carrín M.E., Martini S. (2020). Tailoring physical properties of monoglycerides oleogels using high-intensity ultrasound. Food Res. Int..

[74] Lane K.E., Li W., Smith C., Derbyshire E. (2014). The bioavailability of an omega-3-rich algal oil is improved by nanoemulsion technology using yogurt as a food vehicle. Int. J. Food Sci. Technol..

[75] Gharibzahedi S.M.T., Razavi S.H., Mousavi M. (2015). Optimal development of a new stable nutraceutical nanoemulsion based on the inclusion complex of 2-hydroxypropyl-β-cyclodextrin with canthaxanthin accumulated by Dietzia natronolimnaea HS-1 using ultrasound-assisted emulsification. J. Dispers. Sci. Technol..

[76] Bari A.H., Chawla A., Pandit A.B. (2017). Sono-crystallization kinetics of K2SO4: Estimation of nucleation, growth, breakage and agglomeration kinetics. Ultrason. Sonochem..

[77] da Silva T.L.T., Arellano D.B., Martini S. (2019). Use of high-intensity ultrasound to change the physical properties of oleogels and emulsion gels. J. Am. Oil Chem. Soc..

[78] Sharifi M., Goli S.A.H., Fayaz G. (2019). Exploitation of high-intensity ultrasound to modify the structure of olive oil organogel containing propolis wax. Int. J. Food Sci. Technol..

[79] Giacomozzi A.S., Palla C.A., Carrín M.E., Martini S. (2019). Physical properties of monoglycerides oleogels modified by concentration, cooling rate, and high-intensity ultrasound. J. Food Sci..

[80] Yao Y., Zhou H., Liu W., Li C., Wang S. (2021). The effect of cooling rate on the microstructure and macroscopic properties of rice bran wax oleogels. J. Oleo Sci..

[81] Blake A.I., Marangoni A.G. (2015). The effect of shear on the microstructure and oil binding capacity of wax crystal networks. Food Biophys..

[82] Trujillo-Ramírez D., Reyes I., Lobato-Calleros C., Vernon-Carter E.J., Alvarez-Ramirez J. (2022). Chia seed oil-candelilla wax oleogels structural features and viscoelasticity are enhanced by annealing. LWT.

[83] Jana S., Martini S. (2014). Effect of high-intensity ultrasound and cooling rate on the crystallization behavior of beeswax in edible oils. J. Agric. Food Chem..

[84] Page M.J., McKenzie J.E., Bossuyt P.M., Boutron I., Hoffmann T.C., Mulrow C.D., Shamseer L., Tetzlaff J.M., Akl E.A., Brennan S.E. (2021). The PRISMA 2020 statement: An updated guideline for reporting systematic reviews. Syst. Rev..

[85] Lassila P., Valoppi F., Tommiska O., Hyvönen J., Holmström A., Hietala S., Salmi A., Haeggström E. (2022). Practical scale modification of oleogels by ultrasonic standing waves. Ultrason. Sonochem..

[86] da Silva T.L.T., Danthine S. (2022). Influence of sonocrystallization on lipid crystals multicomponent oleogels structuration and physical properties. Food Res. Int..

[87] Gao Z., Zhang C., Wu Y., Chen F., Hu B., Wang R., Yang J., Nishinari K. (2022). Composite oleogels formed by cellulose particles and sorbitan acid esters. Food Struct..

[88] Jadhav H.B., Pratap A.P., Gogate P.R., Annapure U.S. (2022). Ultrasound-assisted synthesis of highly stable MCT based oleogel and evaluation of its baking performance. Appl. Food Res..

[89] Li L., Taha A., Geng M., Zhang Z., Su H., Xu X., Pan S., Hu H. (2021). Ultrasound-assisted gelation of β-carotene enriched oleogels based on candelilla wax-nut oils: Physical properties and in-vitro digestion analysis. Ultrason. Sonochem..

[90] Szymańska I., Żbikowska A., Kowalska M., Golec K. (2021). Application of Oleogel and Conventional Fats for Ultrasound-assisted Obtaining of Vegan Creams. J. Oleo Sci..

[91] da Silva T.L.T., Danthine S. (2021). Effect of high-intensity ultrasound on the oleogelation and physical properties of high melting point monoglycerides and triglycerides oleogels. J. Food Sci..

[92] Qiu H., Qiu Z., Chen Z., Liu L., Wang J., Jiang H., Zhang H., Liu G.-Q. (2021). Antioxidant properties of blueberry extract in different oleogel systems. LWT.

[93] Valoppi F., Salmi A., Ratilainen M., Barba L., Puranen T., Tommiska O., Helander P., Heikkilä J., Haeggström E. (2020). Controlling oleogel crystallization using ultrasonic standing waves. Sci. Rep..

[94] Liu L., Ramirez I.S.A., Yang J., Ciftci O.N. (2020). Evaluation of oil-gelling properties and crystallization behavior of sorghum wax in fish oil. Food Chem..

[95] Shi Z., Cao L., Kang S., Jiang S., Pang M. (2022). Influence of wax type on characteristics of oleogels from camellia oil and medium chain triglycerides. Int. J. Food Sci. Technol..

[96] Blake A.I., Co E.D., Marangoni A.G. (2014). Structure and physical properties of plant wax crystal networks and their relationship to oil binding capacity. J. Am. Oil Chem. Soc..

[97] Yu Y., Wang T., Gong Y., Wang W., Wang X., Yu D., Wu F., Wang L. (2022). Effect of ultrasound on the structural characteristics and oxidative stability of walnut oil oleogel coated with soy protein isolate-phosphatidylserine. Ultrason. Sonochem..

[98] Omonov T.S., Bouzidi L., Narine S.S. (2010). Quantification of oil binding capacity of structuring fats: A novel method and its application. Chem. Phys. Lipids.

[99] Habibi A., Kasapis S., Truong T. (2022). Effect of hydrogel particle size embedded into oleogels on the physico-functional properties of hydrogel-in-oleogel (bigels). LWT.

[100] Sarkisyan V., Sobolev R., Frolova Y., Vorobiova I., Kochetkova A. (2022). A Study of the Quantitative Relationship between Yield Strength and Crystal Size Distribution of Beeswax Oleogels. Gels.

[101] Sobolev R., Frolova Y., Sarkisyan V., Makarenko M., Kochetkova A. (2022). Effect of beeswax and combinations of its fractions on the oxidative stability of oleogels. Food Biosci..

[102] Wagh A., Birkin P., Martini S. (2016). High-intensity ultrasound to improve physical and functional properties of lipids. Annu. Rev. Food Sci. Technol..

[103] da Silva T.L.T., Danthine S. (2022). High-intensity Ultrasound as a Tool to Form Water in Oleogels Emulsions Structured by Lipids Oleogelators. Food Biophys..

[104] Xia T., Wei Z., Xue C. (2022). Impact of composite gelators on physicochemical properties of oleogels and astaxanthin delivery of oleogel-based nanoemulsions. LWT.

[105] Xia T., Gao Y., Liu Y., Wei Z., Xue C. (2022). Lactoferrin particles assembled via transglutaminase-induced crosslinking: Utilization in oleogel-based Pickering emulsions with improved curcumin bioaccessibility. Food Chem..

[106] Palla C.A., Aguilera-Garrido A., Carrín M.E., Galisteo-González F., Gálvez-Ruiz M.J. (2022). Preparation of highly stable oleogel-based nanoemulsions for encapsulation and controlled release of curcumin. Food Chem..

[107] Yu X.-H., Zhou F.-Z., Xi Y.-K., Huang X.-N., Yin S.-W., Yang X.-Q. (2022). Ethyl cellulose-chitosan complex particles stabilized W/O Pickering emulsion as a recyclable bio-catalytic microreactor. Coll. Surf. A Physicochem. Eng. Asp..

[108] Chen X.-W., Yin W.-J., Yang D.-X., Wan Z.-L., Ma C.-G., Yang X.-Q. (2021). One-pot ultrasonic cavitational emulsification of phytosterols oleogel-based flavor emulsions and oil powder stabilized by natural saponin. Food Res. Int..

[109] Qi W., Li T., Zhang Z., Wu T. (2021). Preparation and characterization of oleogel-in-water pickering emulsions stabilized by cellulose nanocrystals. Food Hydrocoll..

[110] Urbánková L., Sedláček T., Kašpárková V., Bordes R. (2021). Formation of oleogels based on emulsions stabilized with cellulose nanocrystals and sodium caseinate. J. Colloid Interface Sci..

[111] Pehlivanoglu H., Akcicek A., Can A.M., Karasu S., Demirci M., Yilmaz M.T. (2021). Effect of oil type and concentration on solid fat contents and rheological properties of watery oleogels. Riv. Ital. Delle Sostanze Grasse.

[112] Ren Z., Li Z., Chen Z., Zhang Y., Lin X., Weng W., Yang H., Li B. (2021). Characteristics and application of fish oil-in-water pickering emulsions structured with tea water-insoluble proteins/κ-carrageenan complexes. Food Hydrocoll..

[113] Rocha-Guzmán N.E., Cháirez-Ramírez M.H., Pérez-Martínez J.D., Rosas-Flores W., Ornelas-Paz J.D.J., Moreno-Jiménez M.R., González-Laredo R.F., Gallegos-Infante J.A. (2021). Use of organogel-based emulsions (o/w) as a tool to increase the bioaccessibility of lupeol, curcumin, and quercetin. J. Am. Oil Chem. Soc..

[114] Qi W., Zhang Z., Wu T. (2020). Encapsulation of β-carotene in oleogel-in-water Pickering emulsion with improved stability and bioaccessibility. Int. J. Biol. Macromol..

[115] Shi Z., Shi Z., Wu M., Shen Y., Li G., Ma T. (2020). Fabrication of emulsion gel based on polymer sanxan and its potential as a sustained-release delivery system for β-carotene. Int. J. Biol. Macromol..

[116] Pehlivanoglu H., Demirci M., Toker O.S. (2017). Rheological properties of wax oleogels rich in high oleic acid. Int. J. Food Prop..

